# Sepsis-associated acute lung injury: do inflammatory lipid mediators flood the lungs by activating pulmonary TRP channels?

**DOI:** 10.1186/s40635-026-00942-0

**Published:** 2026-07-17

**Authors:** Nada Birkic, Robert R. Ehrman, Đurđica Cekinović Grbeša, Zeljka Minic, Christian A. Reynolds

**Affiliations:** 1 Faculty of Biotechnology and Drug Development, University of Rijeka, Rijeka, Croatia; 2 Department of Emergency Medicine, Wayne State University School of Medicine, Detroit, MI USA; 3 Department of Infectious Diseases, Faculty of Medicine, University of Rijeka, Rijeka, Croatia; 4 Clinic for Infectious Diseases, Clinical Hospital Center Rijeka, Rijeka, Croatia

**Keywords:** Lung injury, Sepsis, Inflammation, Lipid mediators, TRP channels

## Abstract

Although prevalent in critical care, sepsis-associated acute lung injury (sALI) lacks a clear mechanistic framework and disease-modifying therapies. In this narrative review, we synthesize clinical and experimental data implicating lipid mediators (LMs) as underappreciated drivers of pulmonary barrier failure in sALI. LMs are known activators or sensitizers of transient receptor potential (TRP) channels expressed in non-neuronal lung cells. In endothelial and epithelial compartments, activation of TRP channels elevates intracellular Ca²⁺, increases microvascular permeability, impairs alveolar fluid clearance, and promotes pulmonary edema. Converging metabolomic and translational studies associate accumulation of specific LMs with respiratory failure, providing a mechanistic link between the LMs and TRP-dependent barrier dysfunction. We propose the activation of TRP channels by LMs as a key underlying mechanism of sALI. Clarifying this mechanism, biomarker-guided studies may enable more targeted clinical trials and ultimately improve outcomes in sALI.

## Introduction

Sepsis is caused by a dysregulated host response to an infection, resulting in widespread inflammation and multiple organ dysfunction [81]. A global study published in 2020 reported that sepsis affects approximately 49 million people annually and causes 11 million deaths, accounting for 20% of all global mortality. Although the incidence of sepsis decreased between 1990 and 2017, it continues to impose a substantial healthcare and economic burden, particularly in low-income countries [71]. Among the affected organs, the lungs are particularly vulnerable, and sepsis is the most common cause of acute lung injury often referred to as sepsis-associated acute lung injury (sALI) [70, 91]. sALI is a primary driver of the acute respiratory distress syndrome (ARDS) spectrum, and sepsis represents the most common risk factor for ARDS [7, 78, 88].

The current clinical standard for classification of ARDS is the Berlin definition (2012), which categorizes ARDS based on the degree of hypoxemia as measured by the ratio of the partial pressure of oxygen to the fraction of inspired oxygen (*PaO*2/*FiO*2) [66]. A new global definition proposed in 2023 expands these categories to include patients receiving high-flow nasal oxygen (HFNO) and allows for the use of pulse oximetry (*SpO*2/*FiO*2) instead of arterial blood gas measurements (the Kigali modification) [48]. A major goal of critical care research seeks to shift from treating ARDS as a uniform condition to a precision medicine approach that targets specific biological pathways. For decades, critical care trials have been plagued by study cohorts that combine highly heterogeneous etiologies of lung injury, including penetrating traumas, aspiration of gastric contents, pneumonia, and systemic infection under a single umbrella. Pooling such diverse conditions together obscures endotype-specific drivers, producing inconsistent findings, limiting therapeutic translation, and leading to calls for increased endotyping studies in this area [1]. Among the clinical ARDS spectrum hyper-inflammatory and hypo-inflammatory phenotypes are often designated. The recently published PHIND study demonstrated the feasibility of prospectively identifying hyper-inflammatory and hypo-inflammatory ARDS phenotypes based on the combined measurement of IL-6, soluble TNFR1, and arterial bicarbonate [67]. The hyper-inflammatory phenotype is characterized by elevated levels of inflammatory markers and is associated with higher mortality rates. Conversely, the hypo-inflammatory phenotype, is characterized by lower levels of inflammatory markers and is generally associated with better clinical outcomes [110]. However, it remains to be seen how clustering patients by biological features (hyper-inflammatory vs. hypo-inflammatory) will facilitate true precision medicine in ARDS. For instance, pneumonia-associated ARDS may follow a different pathomolecular and clinical trajectory than sepsis-associated ARDS, and we must take care not to obscure these distinctions by phenotype labels [6, 100].

Multiple studies have demonstrated that the plasma metabolomic profile of critically ill ARDS patients differ between survivors and non-survivors [18, 44, 69]. However, it is unknown to what extent plasma metabolic profiling reflects the biology of disease progression. At least 6 prior studies have carried out plasma metabolic profiling in ARDS cohorts [18, 34, 68, 82, 90, 102] and several have identified lipid molecules as key metabolites associated with ARDS diagnosis or severity. It is not surprising that metabolomic profiles of individuals with lung injury secondary to systemic infection (e.g. sALI) differ drastically from those with lung injury secondary to trauma or aspiration, even when clinical phenotypes overlap (i.e. presenting with ARDS). Such heterogeneity aligns with the broader endotyping paradigm proposed for ARDS, where identification of biologically distinct endotypes has been shown to explain divergent outcomes and heterogeneous responses to therapy [13, 83].

Expeditious, accurate recognition and intervention have been cited as one of the greatest barriers to compliance with the recommended standard of care for ARDS [11, 42, 99]. There has been slow but incremental progress in identifying biomarkers that (i) may directly contribute to the pathophysiology of ARDS, (ii) have utility in diagnosis and monitoring, and (iii) are potential therapeutic targets [13]. In this narrative review, we propose a framework implicating inflammatory lipid mediators (LMs) as underappreciated drivers of pulmonary barrier dysfunction in sALI. Among the emerging biochemical pathways linking LMs to sALI, the transient receptor potential (TRP) channels have recently gained substantial attention. We propose a hypothetical model in which specific bioactive LMs (e.g. LA-DiHOMEs) drive sALI via TRP channel activation. This review therefore explores the interplay between LMs and TRP channels in the pathogenesis of sALI. Further investigation into proposed molecular mechanisms may yield more sensitive biomarkers and mechanism-based interventions aimed at improving pulmonary outcomes in sepsis.

## Pathophysiology of sALI

The respiratory system, composed of bronchi, bronchioles, and alveoli, is essential for gas exchange. This function relies on the integrity of the alveolar-capillary barrier, which consists of (i) epithelial cell layer of alveolar type I (AT1) cells which enable gas exchange and type II (AT2) cells which produce surfactants and facilitate repair, (ii) the microvascular endothelial layer, and (iii) the interstitial space between two layers [104, 109]. In sALI, this barrier is predominantly compromised by indirect injury from systemically circulating inflammatory mediators. Once disrupted, barrier dysfunction results in pulmonary edema, impaired gas exchange and progressive respiratory failure. Numerous reviews have comprehensively reported on the various inflammation-induced responses to sepsis [16, 29, 33]; here, we briefly summarize the key events relevant to the development of sALI.

Disruption of the alveolar-capillary barrier during sALI coincides with the rapid activation of the innate immune system. Innate immune cells recognize pathogen-associated molecular patterns (PAMPs) and damage-associated molecular patterns (DAMPs) by pattern recognition receptors (PRRs) [40, 41, 93]. Engagement of these receptors on alveolar macrophages and recruited neutrophils triggers the release of cytokines, chemokines, and LMs to attract more immune cells while activating downstream intracellular signaling pathways. Activation of these pathways triggers the transcription of numerous genes that are involved in the early innate response [43]. Although these responses are essential for pathogen clearance, excessive or prolonged activation causes collateral tissue damage, amplifying epithelial and endothelial injury. As a result, gas exchange is impaired and respiratory symptoms worsen. Moreover, disruption of epithelial ion channels hampers fluid clearance from the alveoli, compounding the gas exchange issues. Ultimately, activation of the innate immune system, accompanied by release of inflammatory mediators, provides the foundation for the molecular storms that drive sALI.

sALI typically results from *indirect* lung injury, where the inciting event is lung endothelial injury in the setting of systemic inflammation. This differs from *direct* lung injuries (e.g. aspiration), which primarily cause lung epithelial injury. Injury to the pulmonary vascular endothelium in sepsis leads to disruption of intercellular junctions and exudation of protein-rich edema fluid into the alveoli. Importantly, preclinical sepsis models effectively replicate clinical sALI findings, including leukocyte infiltration, pulmonary edema and impaired gas exchange (Table 1). Accordingly, these valuable model systems enable novel molecular mechanism to be interrogated and potential disease-modifying biomarkers to be discovered.

**Table 1:** Experimental rodent models of extrapulmonary sepsis and associated pulmonary outcomes

Study	Extrapulmonary sepsis model	Species	Pulmonary outcomes measured	Main observation in lung pathology	Reference
Peralta et al., [62]	Cecal ligation and puncture (CLP)	Rat	Lung MPO, BAL protein, leukocyte oxidative activity in lungs	20× lung MPO rise 6 h post CLP procedure	Peralta et al., [62]
Stamme et al., [87]	Fecal peritonitis (FP) (i.p. injection)	Mouse	BAL cytokines (MCP-1, TNF, IFN-γ), lung MPO, static lung compliance, BAL eicosanoids	Dose-dependent pulmonary inflammation accompanied by decreased lung compliance	Stamme et al., [87]
Fisher et al., [25]	Fecal peritonitis (i.p. injection)	Mouse	Lung MPO, histology score, neutrophil infiltration, lung cytokines, W/D ratio, BAL protein, alveolar fluid clearance	Pulmonary injury marked by inflammation, edema, and loss of epithelial barrier function caused by FP	Fisher et al., [25]
Muniz et al., [54]	Cecal ligation and puncture	Mouse	Lung leukocyte infiltration	Increased leukocyte infiltration into lungs after CLP procedure	Muniz et al., [54]
Zhang et al., [116]	Colon ascendens stent peritonitis (CASP)	Rat	Lung metabolic changes	Lung shows various metabolite shifts during sepsis	Zhang et al., [116]
Fallon et al., [24]	Cecal peritonitis (CP) (i.p. injection)	Mouse (neonates)	Pulmonary edema, neutrophil influx, lung MPO	Marked increase in all measured pulmonary outcomes 24 h after CP procedure	Fallon et al., [24]
Bastarache et al., [5]	Cecal peritonitis (i.p. injection)	Mouse	BAL cells, total BAL protein, W/D ratio, CXCL-1	CP alone induces mild pulmonary alterations, whereas its combination with hyperoxia results in a pronounced acute lung injury	Bastarache et al., [5]
Sharma et al., [77]	Fecal peritonitis (i.p. injection)	Mouse (young vs. aged)	Histological assessment of lung injury, MPO, cytokines in lung tissue homogenates	Elevated MPO and lung cytokine levels (IL-6, IL-10), higher histological injury scores after FP procedure	Sharma et al., [77]
Dibekoğlu et al., [22]	Fecal peritonitis (i.p. injection)	Rat	Histopathological analysis, arterial blood gas analysis, arterial oxygen pressure (PaO_2_) and carbon dioxide pressure (PaCO_2_)	Lung injury and impaired gas exchange caused by FP	Dibekoğlu et al., [22]

Despite growing evidence for the role of bioactive LMs in contributing to sALI, cytokines remain the most extensively studied mediators of sepsis. Excessive release of pro-inflammatory cytokines, including TNF-α, IL-1β, and IL-6, results in endothelial activation, increased vascular permeability, and propagation of tissue damage [64]. In the lungs, these cytokines recruit neutrophils and macrophages, prolong their activation, and amplify oxidative and proteolytic injury. The extent of cytokine release correlates with severity of clinical outcomes. For instance, elevated IL-6 levels are associated with higher mortality [79, 101], while TNF-α and IL-1β directly impair alveolar barrier function [28, 94]. Although anti-inflammatory cytokines such as IL-10 are upregulated in parallel, they are often insufficient to counterbalance the overwhelming pro-inflammatory cytokine storm [115].

**Fig. 1:**
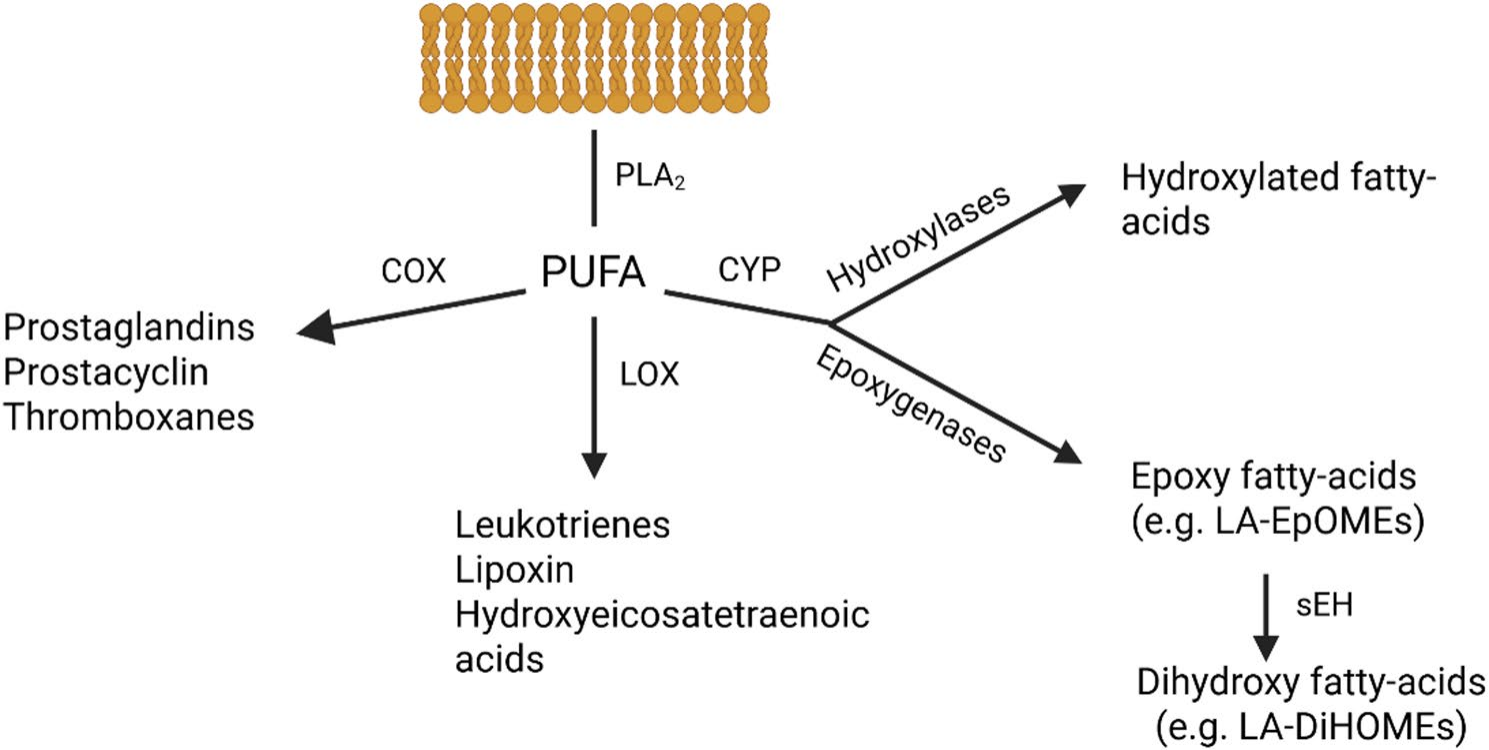
PUFA-derived lipid mediator networks: enzymatic pathways and functional classes. Membrane phospholipids are hydrolyzed by phospholipase A₂ (PLA₂) to release polyunsaturated fatty acids (PUFAs), namely linoleic acid (LA), arachidonic acid (AA), eicosapentaenoic acid (EPA) and docosahexaenoic acid (DHA), which serve as substrates for three major enzymatic routes. The cyclooxygenase (COX) pathway generates prostanoids (prostaglandins and thromboxanes); the lipoxygenase (LOX) pathway yields leukotrienes and hydroxy-eicosanoids and provides precursors for specialized pro-resolving mediators (e.g., lipoxins, resolvins, protectins, maresins); and the cytochrome P450 (CYP) pathway forms ω-hydroxylated products (e.g., 20-HETE) and epoxy-fatty acids (e.g. ETTs), with soluble epoxide hydrolase (sEH) converting epoxides to corresponding diols (e.g. DHETs)

While cytokines have long been considered a hallmark of systemic inflammation, bioactive lipid mediators (LMs) have received comparatively less attention. Yet, growing evidence highlights their crucial role in shaping the immune response. Fatty acyl LMs (e.g. eicosanoids) are endogenous chemical signals that orchestrate responses to infection [21]. These bioactive LMs are derived from polyunsaturated fatty acids (PUFAs), and LM signaling, like cytokine signaling, is extremely complex. Recent advances in mass spectrometry have enabled quantitative identification of numerous novel lipid species and LMs have emerged as important regulators of vascular permeability, interstitial edema, and impaired lung compliance. LMs and their diet-derived PUFA precursors contribute to both pro- and anti-inflammatory states across a wide range of diseases, including ARDS [26, 89]. They are produced through enzymatic oxidation of PUFAs by four families of enzymes: cyclooxygenases, lipoxygenases, epoxygenases, and hydroxylases (Fig. 1) [21]. While cyclooxygenase products have historically dominated the field, since they collectively elicit the cardinal signs of inflammation including fever, swelling, redness, and pain [21], hundreds of additional bioactive LMs are generated during infection [18, 56]. Substantial epoxygenase metabolites can be produced by cytochrome P450 (CYP) isoforms and accumulation of these metabolites is associated with respiratory distress and lung injury [37, 39, 76, 92, 117]. Recent evidence indicates that accumulation of specific CYP-derived LMs, the dihydroxy metabolites of linoleic acid (LA-DiHOMEs), may directly contribute to lung injury and pulmonary dysfunction [32].

Linoleic acid is the primary ω-6 PUFA obtained in the diet and is one of the most abundant fatty acids found in the lipids of cell membranes in individuals consuming a Western diet (Simopoulos A P, 1999) [80]. LA-DiHOMEs arise when linoleic acid is converted by CYP enzymes to epoxy-metabolites of linoleic acid (LA-EpOMEs) in the form of either 9(10)-EpOME or its regioisomer 12(13)-EpOME. Epoxide hydrolase enzymes readily hydrolyze these epoxy acids into corresponding LA-DIHOMEs: 9(10)-DiHOME and 12(13)-DiHOME, respectively. These mono-epoxy fatty acid metabolites of linoleic acid are formed in tissues and are called leukotoxins because they produce toxic effects against leukocytes [39, 92, 117]. In addition to vasodilatory effects, they are also believed to exert toxicity on alveolar epithelial cells and cardiovascular tissues [17, 117]. In accordance with multiple animals’ studies, [51, 76, 92] recent findings from a large retrospective cohort study of sepsis patients identified increased plasma LA-DiHOME concentrations in patients that went on to develop ARDS [68]. Similarly, LA-DiHOME concentrations were found to be highly elevated in COVID-19 patients suffering from ARDS [38, 49].

The term ˝eicosanoid storm˝ has been introduced to describe the massive release of eicosanoids and related LMs such as octadecanoids and docosanoids during infection and inflammation that parallel the well-known cytokine storm [21]. LMs are bioactive signaling lipids derived from PUFAs. The essential PUFAs, alpha linolenic acid (ALA; 18:3n-3) and linoleic acid (LA; 18:2n-6), and their long-chain derivatives, eicosapentaenoic acid (EPA; 20:5n-3), docosahexaenoic acid (DHA; 22:6n-3) and arachidonic acid (AA; 20:4n-6), are the major enzyme substrates for pro and anti-inflammatory LM production (Melissa Gabbs et al., 2015) [50]. LMs derived from cyclooxygenases and lipoxygenases pathways, such as prostaglandins and leukotrienes, are well-established drivers of inflammation and represent classical pharmacological targets exemplified by non-steroidal anti-inflammatory drugs (NSAIDs) and leukotriene receptor antagonists [21]. However, beyond these traditional targets, other LM producing pathways have gained increasing attention. In the case of sALI, various CYP-derived LMs have been implicated in sepsis-associated endothelial dysfunction, vascular leakage, and lung injury. CYP-derived LA metabolites have been shown to induce severe lung injury in experimental models, highlighting their pathogenic potential. Evidence from recent studies indicate that accumulation of the dihydroxy metabolites of LA (LA-DiHOMEs), which are produced in response to systemic infection, directly contribute to lung injury and pulmonary dysfunction. Notably, LA-DiHOMEs directly promote pulmonary capillary leak, interstitial edema, and impaired lung compliance [37, 39, 76, 92, 117]. Consistent with their role in exerting alveolar damage, we hypothesize that accumulation of LA-DiHOMEs directly contributes to lung injury in septic patients. Interesting, although Rogers et al., [68]reported that the global profiling of 970 distinct metabolites in plasma could not fully differentiate patients with early sepsis with ARDS from those without ARDS, the LA-DiHOME, 12,13-DiHOME, was one of 43 metabolites, which included drugs related to intubation and mechanical ventilation (i.e., rocuronium, pantoprazole), that were higher in patients with ARDS, and one of 29 metabolites that was associated with disease severity based on Simplified Acute Physiology Score (SAPS) II.

## TRP channels and their relevance to sALI

Transient receptor potential (TRP) channels are a large superfamily of non-selective cation channels that mediate Ca²⁺ and Na⁺ influx in response to diverse physical and chemical stimuli [15]. Structurally, they are composed of six transmembrane domains with a pore-forming loop between S5 and S6, intracellular N- and C-termini, and assemble as tetramers to form functional ion channels [14]. Based on sequence homology and functional properties, TRPs are classified into six major subfamilies: TRPC (canonical), TRPV (vanilloid), TRPM (melastatin), TRPA (ankyrin), TRPP (polycystin), and TRPML (mucolipin) [114]. Many TRP channels are polymodal, meaning that they can be activated or modulated by different stimuli, including thermal changes, osmotic or mechanical stress, changes in pH, ROS, and exogenous compounds such as capsaicin [15, 98].

While traditionally associated with the activation of sensory neurons, in the respiratory system TRP channels are expressed in various non-neuronal lung cells, including airway epithelial cells, alveolar macrophages, vascular endothelial cells, and smooth muscle cells (Table 2**)**. By regulating intracellular Ca²⁺ levels, TRPs influence key processes such as endothelial permeability [106], epithelial ion transport, cytokine release [63], and immune cell recruitment. Dysregulation of these channels has been linked to multiple lung pathologies, ranging from asthma [12] and COPD [9, 118] to ventilator-induced lung injury [31] and more recently sALI [23].

**Table 2:** TRP channels expressed in non-neuronal (lung) tissue and their location and function

TRP channel	Location in non-neuronal (Lung) tissue	Key roles in lungs
TRPA1	Bronchial epithelial cells, alveolar epithelial cells, fibroblasts, macrophages, neutrophils	Upregulated by inflammatory cytokines in human lung epithelium; responds to chemical irritants/oxidants; contributes to airway inflammation, cough/bronchoconstriction [8, 46, 53, 112]
TRPC1	Lung microvascular endothelial cells	Loss of TRPC1 disrupts adherens junction and induces endothelial hyperpermeability [95]
TRPC6	Pulmonary arterial smooth muscle cells, lung endothelial cells, alveolar macrophages	Regulates Ca²⁺ dependent vascular tone; contributes to vascular permeability [35, 106]
TRPV1	Bronchial fibroblasts, lung epithelial cells, arterial smooth muscle cells, macrophages, neutrophils	Detects inhaled toxicants, inflammatory; promotes neurogenic inflammation and bronchoconstriction [23]
TRPV4	Alveolar epithelial cells, lung endothelial cells, airway smooth muscle cells, macrophages	Regulates endothelial barrier function, contributes to vascular permeability and lung edema [73, 86]
TRPM2	Macrophages	ROS sensor influencing cytokine/chemokine production [52, 65]

TRP family members described below have been implicated in pulmonary dysfunction. The hypothesized involvement of these channels in pathogenesis of sALI is based on experimental studies demonstrating that their pharmacological or genetic inhibition can attenuate pulmonary edema and barrier dysfunction. TRPC6 contributes to vascular tone regulation and neutrophil chemotaxis, with its overactivation increasing endothelial permeability and pulmonary edema [19, 45]. Moreover, selective TRPC6 inhibition, for example with larixyl derivatives, has been shown to mitigate pulmonary edema [106]. Similarly, TRPC6 deletion confers lung protection in experimental rodent models of sepsis [96, 103]. TRPV1, amplifies neurogenic inflammation and cytokine release in the lung microenvironment when activated by toxic inhalants or inflammatory mediators [112]. TRPV4, is central to alveolar–capillary barrier regulation, where its activation disrupts intercellular junctions and promotes edema, whereas inhibition protects against lung injury in experimental models [2]. Finally, TRPA1, though less extensively studied, contributes to airway inflammation and hyperresponsiveness [55].

## Lipid mediators and their modulation of TRP channels

The interaction of inflammatory LMs with TRP channels has most widely been studied in the context of inflammatory pain conditions, due to the predominate expression of these channels on sensory nerves. Table 3 summarizes know LM-TRP channel interactions associated with the activation, inhibition, or sensitization of nociception. While the influence of LMs on nociception via interaction with TRP channels has been well characterized, recent evidence suggests that LM-TRP cannel interactions exert important pathophysiological roles in non-neuronal tissues (e.g. the lung).

**Table 3:** TRP channels and lipid mediators known to modulate their activity

TRP channel	Lipid mediator	Effect	Experimental model	Reference
**TRPV1**	9,10-DiHOME, 12,13-DiHOME, 9(10)-EpoME, 12(13)-EpOME,	Activate	In vitro: Whole-cell patch-clamp and Ca²⁺ imaging in CHO-TRPV1 and rat trigeminal ganglion (TG) neurons In vivo: Mouse burn injury model	Green et al., [30]
	9-HODE, 13-HODE, 9-oxoODE, 13-oxoODE	Activate	In vitro: Ca²⁺ imaging, whole-cell and single cell patch-clamp, in TG and CHO-TRPV1 cells In vivo: Intraplantar injection produced TRPV1-dependent nociceptive behavior and heat hyperalgesia (absent in TRPV1⁻/⁻ mice)	Patwardhan et al., [60], Patwardhan, [61]
	20-HETE	Activate	In vitro: Whole cell patch- clamp, Ca²⁺ imaging in dorsal root ganglion (DRG) neurons and HEK cells	Wen et al., [107]
	5-HETE, 12-HPETE, 15-HPETE, 15-HETE, LTB4	Activate	In vitro: Whole-cell patch-clamp on cultured DRG neurons and VR1-transfected HEK cells	Wook Hwang et al., [111]
	LXA4	Inhibit	In vitro: Ca²⁺ imaging in DRG cells In vivo: TiO₂-induced arthritis mice model	Saraiva-Santos et al., [72]
	RvE1	Inhibit	In vitro: Ca²⁺ imaging, whole-cell patch-clamp in DRG neurons Ex vivo: Patch-clamp in spinal cord slices In vivo: Intrathecal RvE1 administration	Z. Z. Xu et al., [113]
	RvD2	Inhibit	In vitro: Whole-cell patch-clamp recordings in DRG neurons Ex vivo: Spinal cord slices In vivo: Intrathecal RvD2 administration	Park et al., [58], Park et al., [59]
	Maresin 1	Inhibit	In vitro: Whole-cell patch-clamp in TG neurons Ex vivo: Patch-clamp in trigeminal nucleus slices In vivo: intraplantar MaR1 administration	Park et al., [57], Serhan et al., [74]
	Neuroprotectin D1	Inhibit	In vitro: Whole-cell patch-clamp in DRG neurons Ex vivo: Mouse spinal cord slices In vivo: intrathecal NPD1 administration in mice	Park et al., [58]
**TRPV2**	13-HODE	Activate	In vitro: Ca²⁺ imaging in HEK-293 cells overexpressing rat TRPV2	Petrocellis et al., [20]
**TRPV3**	RvD1	Inhibit	In vitro: Ca²⁺ imaging and whole-cell patch-clamp in HEK293T-TRPV3 and HaCaT keratinocytes In vivo: Reduction of TRPV3-mediated heat and chemical pain behaviors	Bang et al., [4]
	17R-RvD1	Inhibit	In vitro: Ca²⁺ imaging and whole-cell patch-clamp in HEK293T-TRPV3 and HaCaT cells In vivo: Intraplantar injection of 17R-RvD1 reversed CFA-induced heat hyperalgesia and FPP-evoked pain	Bang et al., [3]
**TRPV4**	5,6-EpETrE, 8,9-EpETrE	Activate	In vitro: Ca²⁺ imaging and whole-cell/single-cell patch-clamp in TRPV4-transfected HEK-293 cells and primary mouse aortic endothelial cells	Watanabe et al., [105]
	RvD1	Inhibit	In vitro: Ca²⁺ imaging and whole-cell patch-clamp TRPA1 transfected HEK293T cells and DRG neurons	Bang et al., [4]
**TRPA1**	9,10-DiHOME, 12,13-DiHOME, 9(10)-EpoME, 12(13)-EpOME	Activate	In vitro: Whole-cell patch-clamp and Ca²⁺ imaging in transfected CHO-TRPA1 cells and rat TG neurons	Green et al., [30]
	9-HODE, 13-HODE	Activate	In vitro: Ca²⁺ imaging and patch-clamp	Petrocellis et al., [20]
	5,6-EpETrE	Activate	In vitro: Ca²⁺ imaging in mouse DRG neurons Ex vivo: Patch-clamp in spinal cord slices In vivo: Intrathecal 5,6-EpETrE caused TRPA1- dependent mechanical allodynia in mice (absent in TRPA1⁻/⁻ mice)	Sisignano et al., [85]
	LXA4	Inhibit	In vitro: calcium imaging in DRG cells In vivo: TiO₂-induced arthritis model in mice	Saraiva-Santos et al., [72]
	RvD1	Inhibit	In vitro: Ca²⁺ imaging and whole-cell patch-clamp in TRPA1-expressing HEK293T and DRG neurons In vivo: Intradermal RvD1 alleviates TRPA1 channel-mediated acute nociception in mice	Bang et al., [4]

The AA derivatives 12-HpETE, 15-HpETE, 5-HETE, 15-HETE and LTB4, activate TRPV1 channel with similar potencies as it’s natural agonist capsaicin, and this activation is blocked by capsazepine, a TRPV1 antagonist [111]. Another AA metabolite, 20-HETE, not only activates but sensitizes TRPV1 [108]. Interestingly, ω-3 PUFA derivatives such as 20-HEPE (from EPA) and 22-HDoHE (from DHA) display higher efficacy in TRPV1 activation in vitro than 20-HETE yet fail to induce pain behaviors in murine models.

LA derived LMs, 9-HODE, 13-HODE, 9-oxoODE and 13-oxoODE also activate TRPV1 leading to acute pain and thermal hyperalgesia [60]. In vivo, these effects are abolished in TRPV1-deficient mice or by pharmacological antagonists [60]. In addition, both 9-HODE and 13-HODE were reported to activate TRPA1, while 13-HODE has been reported to activate TRPV2 [20]. The epoxy- and dihydroxy- metabolites of LA, 9, 10-DiHOME, 12,13-DiHOME, 9(10)-EpOME and 12(13)-EpOME active both TRPV1 and TRPA1 in vitro and in vivo [30].

Administration of ketoconazole, a general CYP inhibitor, significantly reduced LA-derived DiHOMEs and EpOMEs concentrations in neuronal tissues in a burn injury pain model. Both ketoconazole treatment and TRPV1/TRPA1 antagonism reversed post-burn hypersensitivity, supporting the role of CYP-generated lipid mediators in TRPV1/TRPA1 activation and pain sensitization [30]. Interestingly, three clinical studies have suggested that ketoconazole may be effective in preventing the development of lung injury in high-risk critically ill patients [27, 47, 84]. However, the use of ketoconazole as a treatment for inflammatory respiratory disease was not widely adopted after the Ketoconazole and Respiratory Management in Acute Lung Injury and ARDS (KARMA) Trial, found no significant benefit in a large multicenter clinical trial [97]. However, the KARMA Trial enrolled patients with very heterogeneous lung injury etiologies including, those resulting from penetrating trauma, or aspiration of gastric contents.

Recent molecular dynamics simulations have provided mechanistic insights into how lipid mediators interact with TRPV1 [10]. Birkic et al., showed that several LMs form stable interactions within the vanilloid pocket of TRPV1 and bind in similar manner as capsaicin while others such as 9,10-DiHOME likely act through alternative binding sites or indirect mechanisms to influence TRPV1 activity. Importantly, the free binding energy of various LM within the vanilloid pocket of TRPV1 was higher than that of the canonical agonist, capsaicin.

In contrast to these pro-inflammatory LMs, various anti-inflammatory LMs may act as potent endogenous inhibitors of TRP channels. Resolvin D1 (RvD1) inhibits TRPA1, TRPV3, and TRPV4, attenuating agonist-induced acute pain and reversing hypersensitivity in inflamed tissues [4]. Resolvin D2 (RvD2) emerges as a potent inhibitor of both TRPV1 and TRPA1, abolishing inflammation-induced synaptic plasticity and reversing both acute and chronic inflammatory pain. Resolvin E1 (RvE1), in turn, selectively inhibits TRPV1 and reduces TRPV1-driven pain behaviors [58]. Importantly, maresin 1 (MaR1), a DHA-derived lipid mediator produced by macrophages, also dose-dependently inhibits TRPV1, blocks capsaicin-induced responses, and reduces both inflammation and chemotherapy induced neuropathic pain [75].

Although, various LMs are known to influence nociception via interaction with TRP channels, recent evidence suggests that TRP roles go beyond that. Non-neuronal TRP channels in the lung epithelium and endothelium are directly modulated by eicosanoids, thereby linking LM signaling to barrier integrity. TRPV4 is, for instance, modulated by PUFA metabolites including epoxyeicosatrienoic acids (EETs) [105]. TRPV4 activation by epoxyeicosatrienoic acids increases endothelial permeability and promotes alveolar edema [2, 36].

Takin together, these findings highlight the potential for TRP channels serve as polymodal sensors that integrate inflammatory LM signaling. While much of the evidence derives from nociception and pain models, many of these channels are abundantly expressed in non-neuronal lung cells, where their activation leads to barrier dysfunction and lung edema.

## Critical future investigations

The ultimate goal of ARDS precision medicine is to identify pathomolecularly aligned subgroups of patients that more uniformly respond to a given therapy, and mass spectrometry-based assays will be critical to achieve this goal. Biomarker-guided studies may enable more targeted clinical trials and ultimately improve outcomes in ARDS. To date, most clinical studies rely on (untargeted) global metabolic profiling, which is designed to measure as many metabolites as possible in a given biological sample. This approach is excellent for discovering novel metabolic biomarkers and pathways that may be altered in a patient population. To generate truly clinically actionable data, we must transition to targeted metabolite quantification, which returns absolute concentrations of specific (predefined) metabolites. This will allow for direct comparison between study populations and enable the establishment of clinical baselines. A critical step toward validating the clinical utility of inflammatory LM biomarkers, will involve targeted quantification in clinical populations to assess a dose-response relationships and establish cutoff values.

To accurately assess whether TRP channels and their inflammatory LMs ligands drive alveolar damage in sALI, specific experimental model systems are needed. While TRP channels are widely expressed in non-neuronal lung cells, they are most prominently expressed in sensory neurons. Transgenic animal models that restrict genetic knockout of TRP channels to specific cell types within the lung will enable more precise assessment of our proposed pathomolecular mechanism.

## Conclusions and limitations

Among the clinical ARDS spectrum, hyper-inflammatory and hypo-inflammatory phenotypes are often designated. ARDS is best described as a spectrum of distinct pathophysiological processes culminating in diffuse alveolar damage, and pooling of heterogeneous etiologies (e.g. penetrating traumas, aspiration, pneumonia, and systemic infection) based on broad biological features (hyper-inflammatory vs. hypo-inflammatory), likely obscures underlying pathomolecular drivers of lung injury. While sepsis represents the most common risk factor for ARDS, individual host-responses to systemic inflammation likely impact lung injury severity. Advances in metabolomics and molecular endotyping are poised to identify biomarkers that not only guide patient stratification/enrichment strategies, but that may directly contribute to alveolar damage.

In sepsis, secondary (indirect) lung injury often occurs following activation of the innate immune system and is driven by the accumulation of inflammatory mediators promoting alveolar damage. Here we outline a novel mechanism by which inflammatory LMs, thought their capacity to activate transient receptor potential (TRP) channels, may drive alveolar barrier dysfunction. Thus, we hypothesize that activation of TRP channels within the lung by inflammatory LMs may directly contribute to the development of sALI.

Our proposed molecular mechanism in which circulating inflammatory LMs drive alveolar damage through their interactions with TRP channels has been largely extrapolated from non-sepsis experimental model systems. While associative metabolomic findings in patients suggest that circulating LMs are elevated in sepsis patients that develop lung injury, it remains to be determined if LMs directly contribute to alveolar damage in these patients. TRP channels are non-selective cation channels that are known mediate inflammatory responses and disrupt the alveolar-capillary barrier in models of asthma and COPD, but the extent to which their activation contributes to development of sALI is not well understood. Finally, the interaction of inflammatory LMs with TRP channels has most widely been studied in the context of inflammatory pain conditions and not in models of lung injury. Further investigation into these molecular pathways in the context of sALI could provide rational for novel therapeutic strategies targeting TRP channels or their endogenous LM agonists for improving pulmonary outcomes in sepsis.

## Data Availability

The datasets supporting the conclusions of this review article are referenced within the article.
